# Development of a convenient detection method for *Trichomonas vaginalis* based on loop-mediated isothermal amplification targeting adhesion protein 65

**DOI:** 10.1186/s12879-020-05048-w

**Published:** 2020-05-01

**Authors:** Yuhua Li, Shuai Wang, Haoran Li, Xiaoxiao Song, Hao Zhang, Yujuan Duan, Chengyang Luo, Bingli Wang, Sifan Ji, Qing Xie, Zhenchao Zhang

**Affiliations:** Xinxiang Key Laboratory of Pathogenic Biology, School of Basic Medical Sciences, Xinxiang Medical University, Xinxiang, Henan 453003 People’s Republic of China

**Keywords:** *Trichomonas vaginalis*, Adhesion protein 65, LAMP, Diagnosis, Analytical sensitivity and specificity

## Abstract

**Background:**

Trichomoniasis resulting from *Trichomonas vaginalis* (*T. vaginalis*) has been considered as a commonly seen disease with the transmission way of sex. At present, the detection methods of *T. vaginalis* mainly include wet mount microscopy, culture, PCR, immunofluorescence and ELISA. However, all of these detection methods exist shortcomings.

**Methods:**

In this study, a loop-mediated isothermal amplification (LAMP) assay that targeted the species-specific sequence of adhesion protein 65 (AP65) gene had been conducted to detect *T. vaginalis*. The optimum reaction system and conditions were optimized in this rapid detection method.

**Results:**

The results of sensitivity analysis showed that the LAMP assay targeting the AP65 gene was 1000 times more sensitive than the nested PCR targeting the actin gene commonly used for detection of *T. vaginalis*, and the detecting limitation of the former was 10 trichomonad. Moreover, the amplification of the target gene AP65 by LAMP assay exhibited high specificity and the product was exclusively from *T. vaginalis*. The detection technique of LAMP did not exhibit cross-reactivity with the common pathogens of *Trichinella spiralis*, *Toxoplasma gondii*, *Escherichia coli*, *Candida albicans*, *Staphylococcus aureus*, *Haemophilus*.

**Conclusions:**

According to the present study, the LAMP assay with the target of AP65 gene, was suitable for the early diagnosis of *T. vaginalis* infections. Consequently, the LAMP assay was proposed by the current study as a point-of-care examination and an alternative molecular tool which exhibited the potential value in the treatment, control and prevention of trichomoniasis transmission and relevant complication.

## Background

Trichomoniasis, which is resulting from *T. vaginalis,* a kind of protozoa with flagella, has been considered as a commonly seen curable disease with the transmission approach of sex and the quantity of affected people all around the world reaches 276.4 million [1, 2]. *T. vaginalis* is parasitized in the vagina and urethra of female, and in the urethra and prostate gland of male [3, 4]. The disease is characterized by asymptomatic status with the pathogen and acute or chronic inflammation [5]. As an extracellular parasite, *T. vaginalis* adheres to the surface of epithelial cells, and the damage to epithelial cells mainly depends on direct contact [6]. The wound of the parasitic site of epithelial cells was caused by the mechanical action of pleomorphic pseudopodia of trophozoite passing through and wrapping and flagellum movement, the digestion of releasing hydrolase, phagocytosis, and complex effects of exfoliative factors, after *T. vaginalis* infected human reproductive urinary tract. Then inflammation of tissues and organs was caused [7, 8].

According to statistics, more than 170 million people were infected with the disease every year in the world, and the infection rate of people around the world was different and had an increasing trend [9]. In the United States, nearly 5 million people were infected with *T. vaginalis* every year, while the infection rate was 24.3% in Japan, 23.8% in Uganda and 18.0% in South Africa [10]. In China, the infection rate in different places and people fluctuated greatly, ranging from a few percent to dozens of percent [11–13]. Clinical manifestations of the disease in women possibly contain vulvovaginal irritation, lower abdominal pain, dyspareunia, dysuria, malodorous vaginal discharge [2, 14], although the infection is usually asymptomatic in men or in a few cases with the clinical manifestations of dysuria, irritation, urethral discharge [15, 16]. In addition, pregnant women infected with *T. vaginalis* could cause decrease of birth weights, premature rupture of fetal membrane, premature delivery, abortion [17]. Recent studies have shown that *T. vaginalis* infection is also related to cervical cancer in women [18], prostate cancer in men and infertility [18, 19]. The widespread infection and prevalence of *T. vaginalis* increased the risk of human infection with human immunodeficiency virus (HIV) and mycoplasma, and *T. vaginalis* has become an important co-factor of HIV infection [20].

The accurate diagnosis of trichomoniasis is an important link in the prevention and treatment of the disease, and it is of great significance to prevent the transmission of the disease. At present, the detection methods of *T. vaginalis* mainly contain wet mount microscopy, PCR, culture, immunofluorescence as well as ELISA [21, 22]. Among the diagnostic methods, the wet mount microscopy is low in cost, but poor in sensitivity [23]. The method of culture *T. vaginalis* is widely recognized as the “gold standard” for diagnosing trichomoniasis [24]. The sensitivity of the diagnostic method is high, but it takes a long time and requires high quality specimens, and microscopic examiner needs to be professional and experienced. PCR detection is highly sensitive and specific, but it relies on the expensive instrument, and the amplified products need to be analyzed by gel electrophoresis [25]. The fluor immunofluorescence and ELISA are sensitive, but the specificity is poor, and the cost of antibody is expensive, and the detection time is long [26]. In 2000, Notomi et al. established LAMP technology, which had the advantages of simplicity, rapidity, high sensitivity, strong specificity and low cost [27]. The technology could amplify a large number of target genes in a short time and isothermal condition. In addition, this technology has been applied to the detection of bacteria, viruses and other pathogens [28].

AP65, as a specific protein of *T. vaginalis*, is a dominant functional protein which is targeted both to the surface and hydrogenosomes, and mediate binding to host cells [29, 30]. Garcia et al. indicated that TvAP65 was a prominent adhered protein of trichomonad compared to other adhesin, and the protein was the hydrogenosomal NAD-dependent decarboxylating malic enzyme [31]. In this study, we developed a novel way to detect *T. vaginalis* based on loop-mediated isothermal amplification targeting adhesion protein 65.

## Methods

### Parasites

In the current work, the strain of *T. vaginalis* was isolated from the vaginal secretions of female patients clinically exhibiting certain trichomoniasis symptoms in the Third Affiliated Hospital of Xinxiang Medical University. The culture of *T. vaginalis* was performed using 10% calf serum, TYM medium added with antibiotics (100 mg/ml ceftriaxone, 50 mg/ml ciprofloxacin), fungicides (2.5 mg/ml amphotericin B) in a humidified chamber containing 5% CO_2_ at 37 °C. The stationary phase parasites (2 × 10^6^ parasites) were collected using a centrifuge and used subsequently in the experiments.

### DNA extraction of *T. vaginalis*

PBS (pH 7.2) was used to wash the *T. vaginalis* trophozoite for thrice, which was subsequently isolated by centrifugation under the velocity of 5000 rpm for 5 minutes and subjected to DNA extraction using commercial kits (OMEGA) in accordance with the instruction.

The DNA simple of *Trichinella spiralis*, *Toxoplasma gondii*, *Escherichia coli*, *Candida albicans*, *Staphylococcus aureus*, *Haemophilus* were stored in Xinxiang Key Laboratory of Pathogenic Biology.

### Detection of actin gene from *T. vaginalis* using nest-PCR

DNA extracts were subjected to species-specific nested PCR for *T. vaginalis* actin (GenBank Accession No. AF237734). Briefly, the primary PCR was performed in a 25 μL reaction mixture consisting of 12.5 μL of 2 × PCR Master Mix (YIFEI XUE BIOTECHNOLOGY, Nanjing, China), 1 μL (0.1 μmol/L) each of the primers OUT-F and OUT-R (Table 1), 1.0 μL of DNA template (100-200 ng), and 9.5 μL of double distilled water. The procedures and conditions of amplifying DNA included: 3 minutes at 95 °C for denaturation, 95 °C for 45 s, 55 °C for 30 s, and 72 °C for 1 min through 35 cycles, and 72 °C for 10 min for final extension. Then the second PCR was performed as the primary PCR, and 1.0 μL the products of primary PCR and 1 μL (0.1 μmol/L) each of the primers IN-F and IN-R (Table 1) were used as template and primers in this reaction. Electrophoresis using 1.5% agarose stained by 1.0 μL of Goldview Safe DNA Gel Stain (Yifei Xue Biotechnology, Nanjing, China) in advance was used to resolve the obtained DNA sequences at 100 V and a UV transilluminator (UVP, Upland, CA, USA) was used to visualize the bands.

**Table 1:** Oligonucleotide primer sequences used for Nested PCR in this research

Name	Sequences(5′-3′)	Description
OUT-F	TCTGGAATGGCTGAAGAAGACG	Forward primer for the actin gene of *T. vaginalis* in the first stage
OUT-R	CAGGGTACATCGTATTGGTC	Reverse primer for the actin gene of *T. vaginalis* in the first stage
IN-F	CAGACACTCGTTATCG	Forward primer for the actin gene of *T. vaginalis* in the second stage
IN-R	CGGTGAACGATGGATG	Reverse primer for the actin gene of *T. vaginalis* in the second stage
AP65-FIP	GCCGACATAGAAGGATGGGA CGCCCACTCAACCCAAAGGC	Forward inner primer for the AP65 gene of *T. vaginalis* in LAMP assay
AP65-BIP	CCTCTACTCCTCTGGCCGTACAA ACTGTGTGGGAAACACCAT	Backward inner primer for the AP65 gene of T. vaginalis in LAMP assay
AP65-F3	CAACAGAGCACCCAGTTCTT	Forward outer primer for the AP65 gene of T. vaginalis in LAMP assay
AP65-B3	TGTGGAAGGGAGTAGCCTT	Backward outer primer for the AP65 gene of T. vaginalis in LAMP assay

### Designing the primers of LAMP

LAMP Designer version 1.02 (Premier Biosoft, Palo Alto, CA, USA) was used to design the specific LAMP primers of *T. vaginalis*AP65 on the basis of the sequence in NCBI (GenBank Accession No. U35243.1). Four primers including outer primers (AP65-F3, AP65-B3), inner and forward primers (AP65-BIP, AP65-FIP) were chosen (Table 1). BLAST search was used to assess the specificity of these primers towards the sequences of target genes recorded by NCBI (http://www.blast.ncbi.nlm.nih.gov).

### Detection of AP65 gene of *T. vaginalis* by LAMP assays

The mixed reaction solution with the volume of 20 μL which contained Betaine (5 M) 6 μL (Sigma, St. Louis, MO, USA), 10 × Bst DNA polymerase Buffer 2 μL, dNTPs Mixture (10 mM each) 2 μL, each of AP65-FIP and AP65-BIP (16 μM) 1 μL, each of F3 and B3 (4μM) 1 μL, MgCl_2_ (25 mM) 3 μL, 8.0 U Bst DNA polymerase (TaKaRa, Dalian, China) 1.5 μL, and ddH_2_O 1.5 μL was used to perform the LAMP assays. The temperature condition of the LAMP reaction system was 63 °C for 120 min during the incubation and 80 °C for 10 min during the termination in a heating block. The experiment contained negative control (ddH_2_O) and positive control (*T. vaginalis* DNA).

The LAMP products were assessed by gel electrophoresis and addition of SYBR GreenI(Solarbio, Beijing, China). For gel electrophoresis, 1.5% agarose gel was used to resolve 5.0 μL of the product of LAMP, as mentioned above, and the positive result was indicated by the pattern of bands like ladders. For addition of SYBR GreenI, 2.0 μL of 1000× SYBR GreenIwas added to the remainder 15 μL of LAMP products, and the positive result was revealed by dull to fluorescent signal or change of colors from orange to green under visible light.

### Analytical sensitivity of detection of AP65 gene of *T. vaginalis* by LAMP assay

The DNA with the initial concentration of 90 ng/μL was diluted by 10^1^–10^12^ times and kept for use. The trophozoites of *T. vaginalis* were diluted to 10^3^, 10^2^, 10^1^ and 1 respectively, and the DNA of these diluted samples was extracted. Then, the prepared DNA samples were amplified by nested PCR and LAMP respectively, as described above. All the experiments were conducted in triplicate independently.

### Analytical specificity of detection of AP65 gene of *T. vaginalis* by LAMP assay

The examination of the analytical specificity of AP65 gene of *T. vaginalis* by LAMP assay was conducted for determining the cross-reactive degree among DNA extraction samples from various trichomonads, *Trichinella spiralis*, *Toxoplasma gondii*, *Escherichia coli*, *Candida albicans*, *Staphylococcus aureus* and *Haemophilus* with close relation. The experiment contained negative control (ddH_2_O) and positive control (*T. vaginalis* DNA). All the experiments were conducted in triplicate independently.

## Results

### Detecting *T. vaginalis* by nested PCR and LAMP

**Fig. 1:**
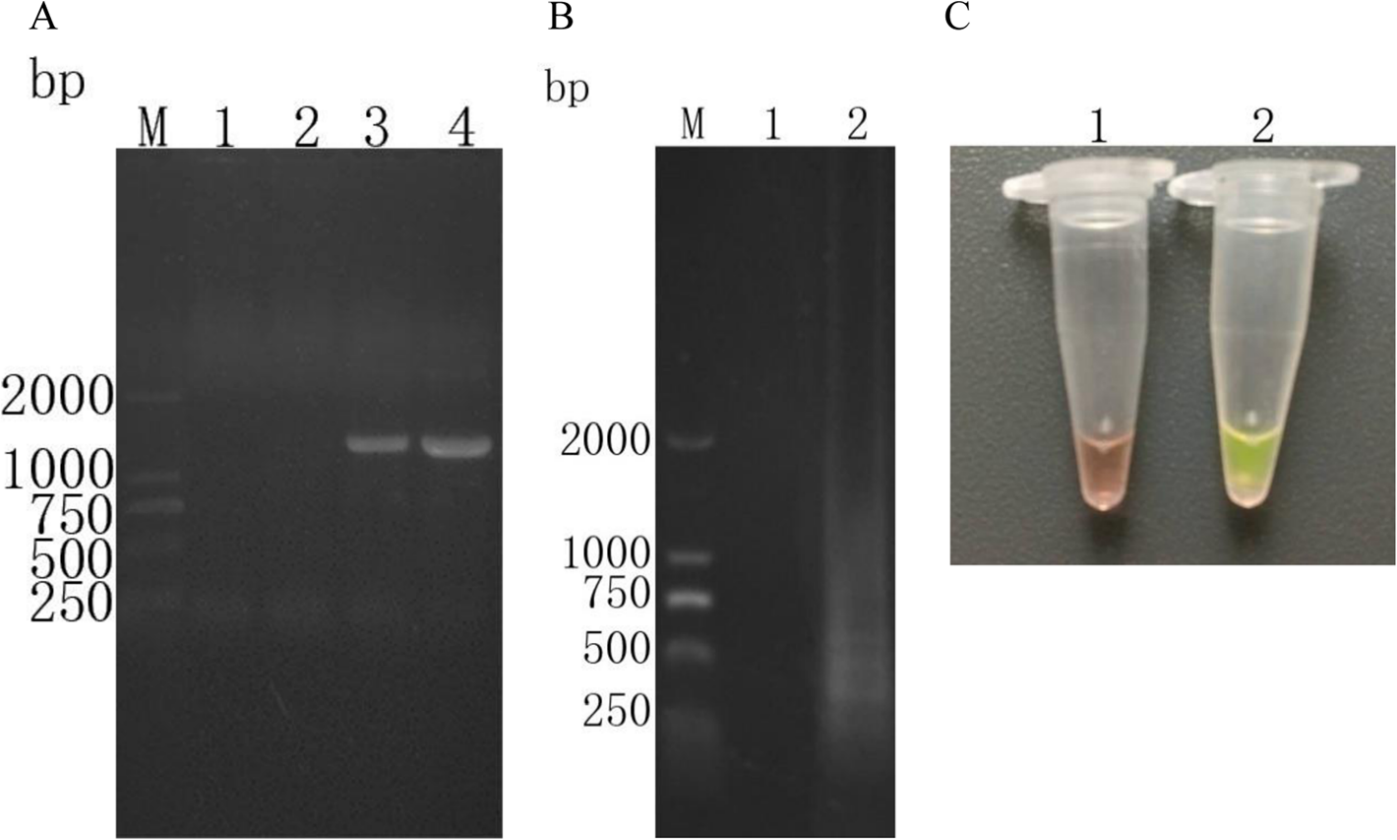
Detection of *T. vaginalis* with amplification of actin gene by nested PCR and AP65 gene by LAMP. **a**: Agarose gel electrophoresis of nested PCR products. (Lane M) DL 2000 the DNA molecular weight marker (ordinate values are expressed in bp); (Lane 1) Negative control outer amplification products; (Lane 2) Negative control inner amplification products; (Lane 3) *T. vaginalis* DNA outer amplification products; (Lane 4) *T. vaginalis* DNA inner amplification products. **b**: Agarose gel electrophoresis of LAMP products. (Lane M) DL 2000 the DNA molecular weight marker (ordinate values are expressed in bp); (Lane 1) Negative control LAMP products; (Lane 2) *T. vaginalis* DNA LAMP products; **c**: LAMP products detected by addition of SYBR GreenI. (Lane 1) Negative control LAMP products (orange); (Lane 2) *T. vaginalis* DNA LAMP products (green)

*T. vaginalis* actin gene was amplified using nest PCR with specific primers, and a positive sequence of about 1100 bp was obtained in line 3 and 4 with the expectation (Fig. 1a). There were no target amplicons in negative controls. The products of nest PCR were further confirmed by sequence analysis.

According to the results of electrophoresis, the products amplified by LAMP with the target of AP65 in *T. vaginalis* exhibited distinctive pattern of multiple bands (Fig. 1b). An orange-to-green color change was observed under natural light or a dull to fluorescent signal under UV transilluminator, after addition of SYBR GreenI(Fig. 1c).

### The analytical sensitivity of *T. vaginalis* nested PCR and LAMP

**Fig. 2:**
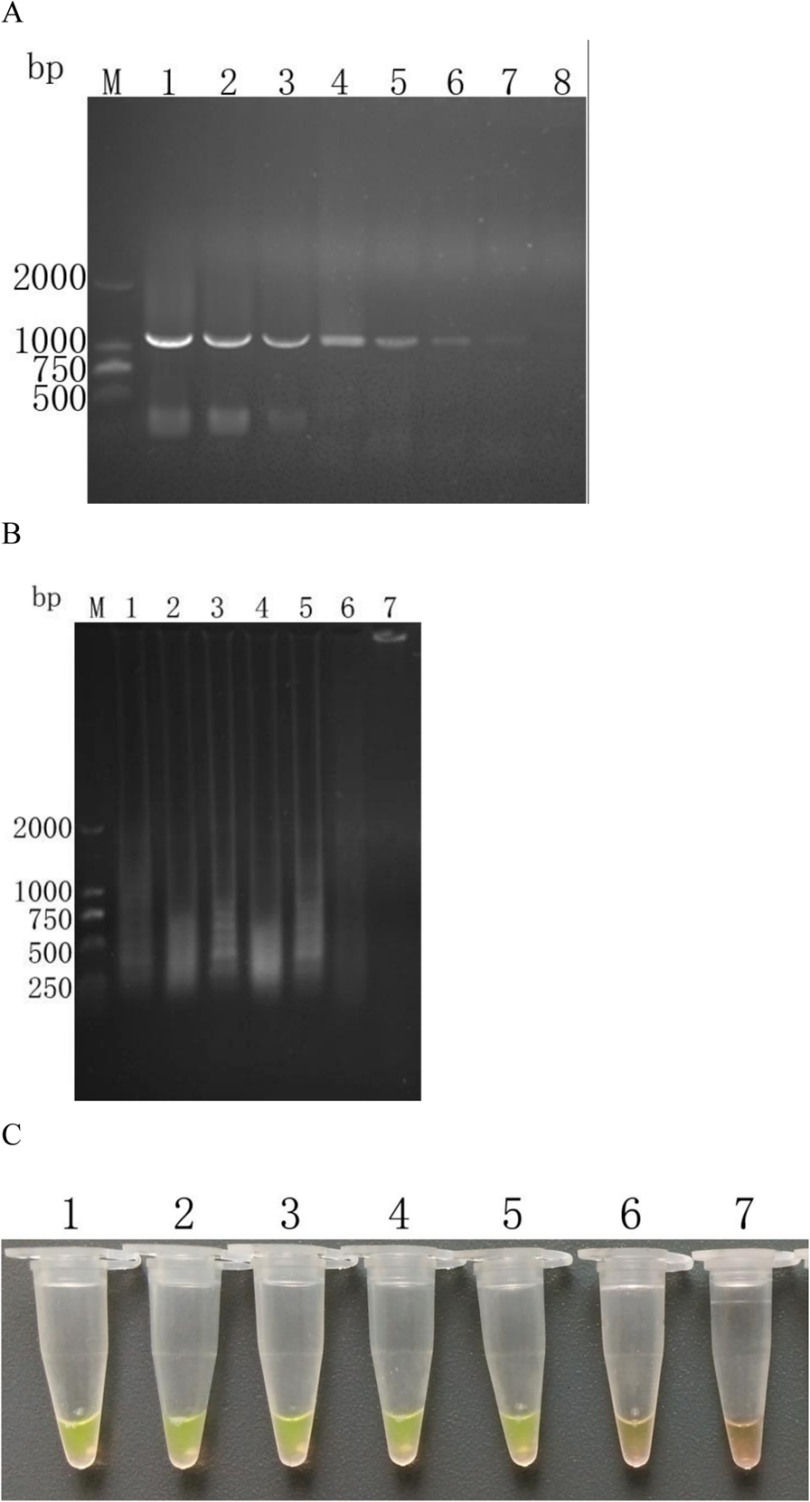
Compare sensitivity of detection of *T. vaginalis* by nested PCR and LAMP with gradient dilution of *T. vaginalis* DNA template. **a**: Agarose gel electrophoresis of inner amplification products.(Lane M) DL 2000 the DNA molecular weight marker (ordinate values are expressed in bp); (Lane 1–8) The initial concentration of 90 ng/μL of *T. vaginalis* DNA was diluted by 10^1^–10^8^ times, amplified by outer amplification, and then amplified by inner amplification. **b**: Agarose gel electrophoresis of LAMP products. (Lane M) DL 2000 the DNA molecular weight marker (ordinate values are expressed in bp); (Lane 1–7) The initial concentration of 90 ng/μL of *T. vaginalis* DNA was diluted by 10^6^–10^12^ times. **c**: LAMP products detected by addition of SYBR GreenI. (Lane 1–7) The initial concentration of 90 ng/μL of *T. vaginalis* DNA was diluted by 10^6^–10^12^ times

The initial concentration of 90 ng/μL of *T. vaginalis* DNA was diluted by 10^1^–10^12^ times. As shown in Fig. 2, the lowest template concentration was 90 × 10^− 7^ ng/μL detected by nested PCR (Fig. 2a), while that was 90 × 10^− 10^ detected by LAMP amplification (Fig. 2b and c). It could be seen that the analytical sensitivity of LAMP assays of *T. vaginalis* established in this study was 1000 times higher than that of nested PCR.

**Fig. 3:**
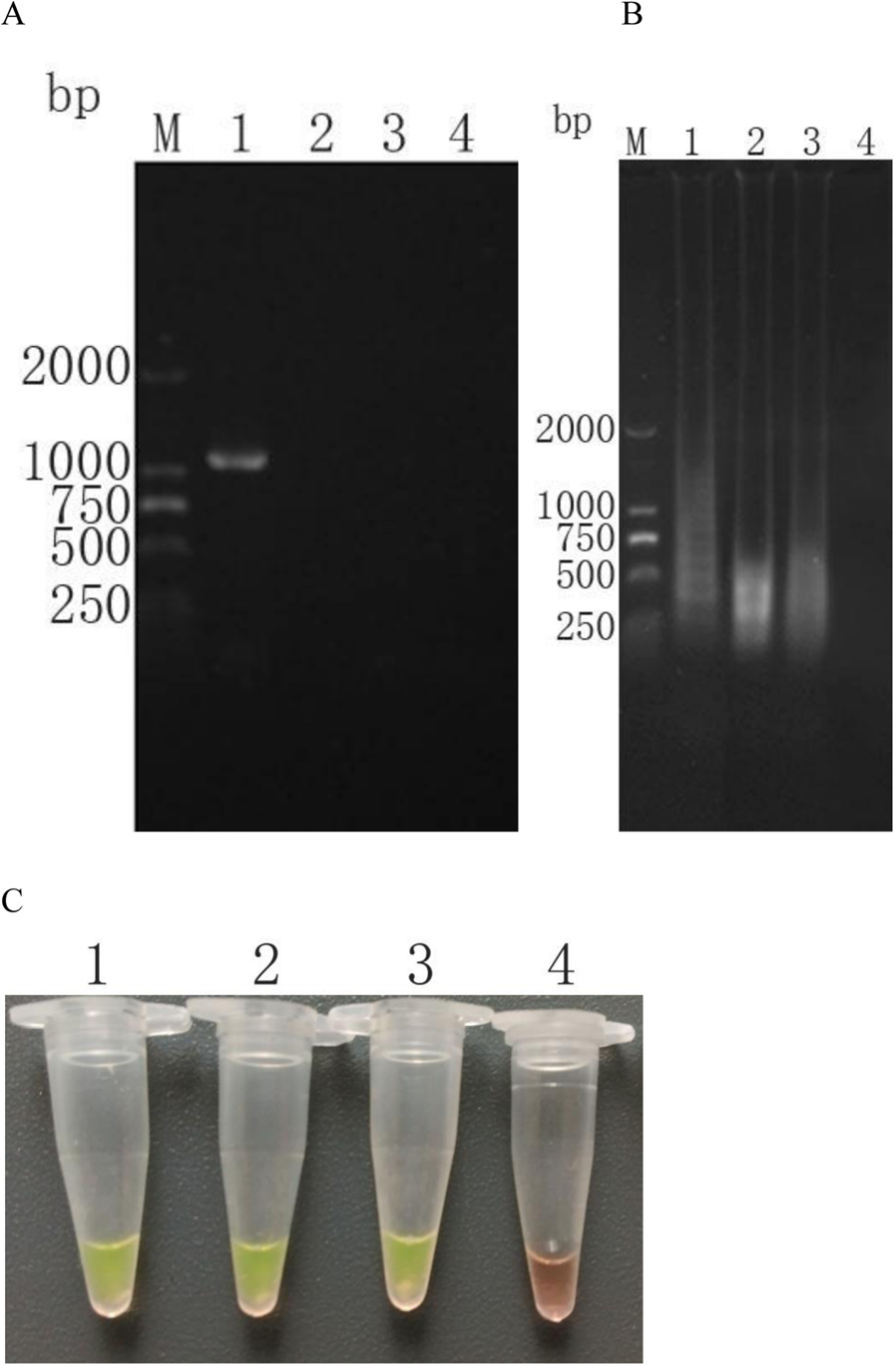
Compare sensitivity of detection of *T. vaginalis* by nested PCR and LAMP with gradient dilution of *T. vaginalis* trophozoites. **a**: Agarose gel electrophoresis of inner amplification products. (Lane M) DL 2000 the DNA molecular weight marker (ordinate values are expressed in bp); (Lane 1–4) The *T. vaginalis* trophozoites were diluted to 10^3^, 10^2^, 10^1^ and 1, amplified by outer amplification, and then amplified by inner amplification. **b**: Agarose gel electrophoresis of LAMP products. (Lane M) DL 2000 the DNA molecular weight marker (ordinate values are expressed in bp); (Lane 1–4) The *T. vaginalis* trophozoites were diluted to 10^3^, 10^2^, 10^1^ and 1. **c**: LAMP products detected by addition of SYBR GreenI. (Lane 1–4) The *T. vaginalis* trophozoites were diluted to 10^3^, 10^2^, 10^1^ and 1

In order to further explore the sensitivity of LAMP amplification, the trophozoites of *T. vaginalis* were diluted to 10^3^, 10^2^, 10^1^ and 1 respectively, and the DNA of these diluted samples was extracted. These results were shown that 10^3^ trophozoites of *T. vaginalis* could be detected by nested PCR (Fig. 3a), while 10 trophozoites of *T. vaginalis* could be detected by LAMP amplification (Fig. 3b and c).

### The analytical specificity of LAMP for *T. vaginalis*

**Fig. 4:**
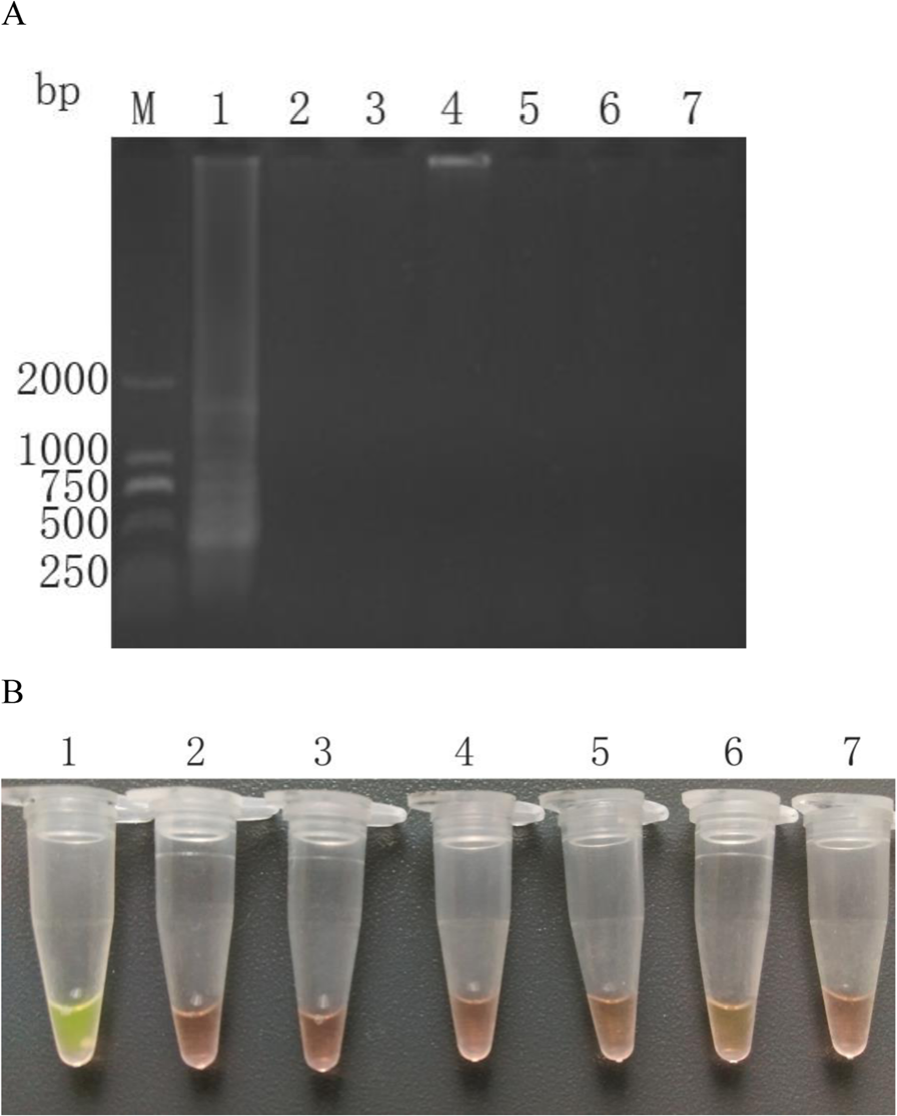
Analytical specificity of detection of *T. vaginalis* by LAMP. **a**: Agarose gel electrophoresis of LAMP products. (Lane M) DL 2000 the DNA molecular weight marker (ordinate values are expressed in bp); (Lane 1–7) The template DNA for LAPM amplification was from *T. vaginalis*, *Trichinella spiralis*, *Toxoplasma gondii*, *Escherichia coli*, *Candida albicans*, *Staphylococcus aureus* and *Haemophilus* respectively. **b**: LAMP products detected by addition of SYBR GreenI. (Lane 1–7) The template DNA for LAPM amplification was from *T. vaginalis*, *Trichinella spiralis*, *Toxoplasma gondii*, *Escherichia coli*, *Candida albicans*, *Staphylococcus aureus* and *Haemophilus* respectively

There were no cross reactions in the LAMP using DNA extractions from *T. vaginalis* and other microorganisms including *Trichinella spiralis*, *Toxoplasma gondii*, *Escherichia coli*, *Candida albicans*, *Staphylococcus aureus* and *Haemophilus*. There were objective bands exclusively in experiments using DNA from *T. vaginalis,* either the results were observed under visible light by adding SYBR GreenIor using a UV transilluminator (Fig. 4).

## Discussion

As a kind of protozoan parasite that infects the genito-urinary system of human beings, *T. vaginalis* is found worldwide but receives less attention compared to other agents of STIs such as *Neisseria gonorrhoeae* and *Chlamydia trachomatis* [32–35]. From 2011 to 2014, the *T. vaginalis* prevalence in Granada, a capital city in Southern Spain was 2.4% [36]. According to the results of a cross-sectional research in a city attached to Yunnan Province, southern China, the *T. vaginalis* prevalence was 9.0% among 734 female sex workers [37]. In addition, according to an observation conducted in Xinxiang of Henan Province, China, 267 of 16,294 (1.64%) samples from female patients suffering from certain trichomoniasis symptoms exhibited clinical positive results under wet mount microscopy [11].

Accurate and rapid diagnosis of trichomoniasis is the key process of treatment, prevention and blocking transmission. However, there are disadvantages in the existing diagnostic methods [14]. In this study, the developed LAMP assay was a rapid and handy way to detect *T. vaginalis* with high analytical sensitivity and specificity, which was verified as superior to traditional nested PCR. Complicated equipment like thermal cycler was not necessary for LAMP, which could be conducted using hot water bath or common heating block. Moreover, the time for producing 10^9^ target gene copies by the LAMP assay was no more than 3 h, which could be reduced by half through the usage of additional loop primers, which was perfect for the point-of-care examinations demanding rapidness [27]. It was revealed by an orange-to-green color change due to adding SYBR GreenIthat large quantity of target gene copies were amplified successfully using LAMP [38]. The LAMP has been considered as a tool of cost-efficiency and rapidness for detecting numerous infections like HIV and malaria, although the potential value of which in detecting *T. vaginalis* hasn’t been developed up to now [39].

Previous researches have shown that LAMP has a 10 to 1000 fold lower limit of detection compared to conventional PCR [40–42]. A LAMP assay designed to detect *Cyrptosporidium parvum* was even proven to have a 100,000 fold lower limit of detection compared to PCR [43]. The LAMP assay exhibited 1000 times higher sensitivity than the nested PCR in amplifying target genes of *T. vaginalis*. According to the results, the detecting limitation of LAMP was 10 trophozoites of *T. vaginalis* and that of nested PCR was as many as 10^3^, and the difference was possibly due to the *Bst* DNA polymerase which was highly tolerate to inhibitors of nucleic acid amplification tests. Toye et al. indicated that PCR was rarely inhibited using urine specimens, infrequently inhibited using endocervical swabs while frequently inhibited using urethral swabs [44]. Although the inhibiting effect could be reduced by various preparing approaches, it is not sufficient to eliminate the inhibitors of PCR from genital swabs by boiling the specimens. Moreover, for LAMP there was no decrease of signal intensity up to 100 trichomonads/mL and the bands in gel with stains of SYBR Safe exhibited typical ladder-like pattern, possibly resulting from significantly higher amplifying product quantity in comparison to that of PCR [38]. As complicated body fluid, urine and genital secretion had been applied to detecting *T. vaginalis* on the basis of nucleic acid, and the sensitivity of which were 64–100% for urine samples and 81–100% to genital secretion swabs [45, 46]. Previous studies have shown that LAMP reactions were not inhibited in spiked urine specimens [47]. Nevertheless, due to the usage of the wild type enzyme in the LAMP approach, unwanted activity of DNA polymerase in the process of setup possibly led to fail in reproducible amplification. In order to solve this problem, a warm start strand-displacing DNA polymerase which kept stable under ambient temperature while didn’t work until 50 °C was used by Tanner et al. [48]. In our study, the time cost for performing LAMP of *T. vaginalis* with high analytical sensitivity from preparing samples to detecting LAMP products was no longer than 130 min.

Although some studies showed that LAMP could detect trichomonad based on target genes of actin, 18S rRNA and the 2-kbp repeated DNA, these genes have relatively high homology among different species [49–51]. AP65 is a prominent adhesin of *T. vaginalis* that mediates binding of parasites to host vaginal epithelial cells (VECs) [52]. We found that the sequences of AP65 had almost no homology with other species. Accordingly, AP65 as the target gene for detection possessed excellent specificity. In this current study, the LAMP of *T. vaginalis* was highly specific without crossreactivity to trichomonads of human beings with close relationship, such as *Trichinella spiralis*, *Toxoplasma gondii*, *Escherichia coli*, *Candida albicans*, *Staphylococcus aureus* and *Haemophilus*. Many previous studies shown that 6 primers were used for LAMP amplification of target genes [53, 54]. However, in the research, the 4 primers that recognized 6 instinct sites of the target gene led to the high specificity of LAMP, and AP65 gene could be detected specifically and sensitively without primers LF and LB. Therefore, the AP65 gene of *T. vaginalis* which was selected as the target gene for conducting highly specific LAMP could be used in the diagnosis of trichomoniasis [55, 56].

Nevertheless, there are a few shortages of LAMP. In spite of the high sensitivity and specificity, it is possible for the large quantity of DNA sequences obtained through the experiment to be contaminated by the opened tubes, which results in fault-positive consequences. In most cases, staining materials of nucleic acids, such as SYBR GreenIand Pico Green, are used in LAMP for detecting the product, while these materials exert an inhibiting effect on the amplifying process of LAMP. In addition, more than 1% contamination of biological substances such as blood and urine or more than 30% of saline solution or PBS can inhibit LAMP as well as PCR reactions [57]. Raw milk contaminants can even inhibit LAMP but not PCR. Consequently, using a real-time turbimeter to monitor the reaction in close tubes [38], using hydroxylnaphthol blue dye added in advance to conduct colorimetric determination [58], or adding wax barrier to SYBR GreenI [59] are capable of preventing contamination due to opened tubes for visualizing endpoint results of LAMP.

## Conclusions

The LAMP assay of *T. vaginalis* based on AP65 gene in the current work is convenient, specific, sensitive, and rapid way to detect trichomoniasis. However, this is a basic research, and more studies are needed in the future for verifying the diagnosing value of the LAMP assay of *T. vaginalis* in clinic. The valid and costeffective nucleic acid amplification test exhibits a promising potential in accurately diagnosing, extensively monitoring, effectively controlling and screening trichomoniasis in resource-limited and point-of-care settings.

## Data Availability

All of the data in the present research are contained in the article.

## Abbreviations

TV: *Trichomonas vaginalis* (*T. vaginalis*)
LAMP: Loop-mediated Isothermal Amplification
AP65: Adhesion Protein 65
PCR: Polymerase Chain Reaction
HIV: Human Immunodeficiency Virus
PBS: Phosphate Buffer Saline
ELISA: Enzyme Linked Immunosorbent Assay
TYM: Trypticase Yeast extract Maltose
NCBI: National Center for Biotechnology Information.
